# Combined Intraperitoneal and Intrathecal Etanercept Reduce Increased Brain Tumor Necrosis Factor-Alpha and Asymmetric Dimethylarginine Levels and Rescues Spatial Deficits in Young Rats after Bile Duct Ligation

**DOI:** 10.3389/fncel.2016.00167

**Published:** 2016-06-23

**Authors:** Jiunn-Ming Sheen, Yu-Chieh Chen, Mei-Hsin Hsu, You-Lin Tain, Hong-Ren Yu, Li-Tung Huang

**Affiliations:** ^1^Department of Pediatrics, Kaohsiung Chang Gung Memorial Hospital and Chang Gung University College of MedicineKaohsiung, Taiwan; ^2^Graduate Institute of Clinical Medical Sciences, Chang Gung University College of MedicineKaohsiung, Taiwan

**Keywords:** asymmetric dimethylarginine, bile duct ligation, etanercept, intrathecal, spatial memory, tumor necrosis factor-α, young age

## Abstract

**Background**: Rats subjected to bile duct ligation (BDL) exhibit increased systemic oxidative stress and brain dysfunction characteristic of hepatic encephalopathy (HE), including fatigue, neurotransmitter alterations, cognitive and motor impairment, and brain inflammation. The levels of tumor necrosis factor-alpha (TNF-α) and asymmetric dimethylarginine (ADMA) are both increased in plasma and brain in encephalopathy induced by chronic liver failure. This study first determined the temporal profiles of TNF-α and ADMA in the plasma, brain cortex, and hippocampus in young BDL rats. Next, we examined whether etanercept was beneficial in preventing brain damage.

**Methods**: Young rats underwent sham ligation or BDL at day 17 ± 1 for 4 weeks. Treatment group rats were administered etanercept (10 mg/kg) intraperitoneally (IP) three times per week with or without etanercept (100 μg) intrathecally (IT) three times in total.

**Results**: We found increased plasma TNF-α, soluble tumor necrosis factor receptor 1 (sTNFR1), soluble tumor necrosis factor receptor 2 (sTNFR2), and ADMA levels, increased cortical TNF-α mRNA and protein and ADMA, and hippocampal TNF-α mRNA and protein, and spatial defects in young BDL rats. The increase in cortex *TNF-α* mRNA and ADMA were reduced by IP etanercept or combined IP and IT etanercept. Dually IP/IT etanercept administration reduced the increased cortical and hippocampal TNF-α mRNA and protein level as well as spatial deficits.

**Conclusions**: We conclude that combined intraperitoneal and intrathecal etanercept reduce increased brain TNF-α and ADMA levels and rescues spatial deficits in young rats after BDL.

## Background

Hepatic encephalopathy (HE) is a complex neuropsychiatric syndrome present in patients with liver disease that is characterized by personality changes, diminished intellectual capacity, motor incoordination, and consciousness disturbance (Collie, 2005; Häussinger and Sies, 2013). Bile duct ligation (BDL) in rats can result in chronic liver failure with accompanying brain dysfunction that are characteristic of HE, including changes in neurotransmitters (Celik et al., 2005; Dhanda and Sandhir, 2015), cognitive and motor impairment (Huang et al., 2009; Magen et al., 2010), and brain inflammation (Rodrigo et al., 2010).

Tumor necrosis factor-alpha (TNF-α) is a central proinflammatory cytokine, with pleiotropic effects on inflammatory and immunological processes (McCoy and Tansey, 2008; Clark et al., 2010). Circulating levels of TNF-α are not only increased in patients with chronic liver failure but also correlate significantly with the severity of HE (Odeh et al., 2004; Jain et al., 2013). Animal studies also show increased TNF-α levels in acute (Chastre et al., 2012; D’Mello et al., 2015) and chronic liver failure (Balasubramaniyan et al., 2012). Odeh proposes that TNF-α plays a central role in the pathogenesis of HE associated with both acute and chronic liver failure (Odeh, 2007).

Asymmetric dimethylarginine (ADMA) is a naturally occurring amino acid that can competitively inhibit nitric oxide synthase (NOS), thereby decreasing the synthesis of NO (Vallance et al., 1992). ADMA and NO are both involved in cognition (Edwards and Rickard, 2007; Miralbell et al., 2013). Bajaj et al. (2013) found that patients with liver cirrhosis had poor cognition and higher serum ADMA. Similarly, young BDL rats exhibit increased circulating and hepatic ADMA levels as well as cognition deficits (Huang et al., 2010; Sheen et al., 2014).

A previous study showed that treatment of cultured rat fibroblast-like synoviocytes with TNF-α significantly increased the levels of ADMA and decreased the expression of dimethylarginine dimethyl-aminohydrolase 2 (DDAH2) mRNA and protein (Chen et al., 2013); these effects of TNF-α were abolished by DDAH2 overexpression (Chen and Zhang, 2011). ADMA has been implicated in the inflammatory response and production of TNF-α in cultured endothelial cells (Chen et al., 2007). Moreover, anti-TNF-α therapy may improve the plasma L-arginine/ADMA ratio in patients with inflammatory arthropathies (Angel et al., 2012).

Etanercept is a dimeric recombinant form of the extracellular domain of the human p75 TNF-α receptor 2 fused to the Fc fragment of human immunoglobulin G1 (Banks et al., 1995; Wong et al., 2008). Peripheral systemic etanercept administration can delay the progression of azoxymethane-induced HE in C57BL/6 mice by reducing hepatocellular damage and decreasing both systemic and central inflammation (Chastre et al., 2012). In addition, perispinal administration of etanercept has been shown to be an effective therapy for brain disorders (Tobinick, 2009, 2010). However, etanercept does not cross the blood-brain barrier (BBB) when administered systemically (Banks et al., 1995). Few studies have used the intrathecal/intraventricular route to deliver an anti-TNF-α drug for treating brain disease (Heldmann et al., 2005; Riazi et al., 2008; Medeiros et al., 2010; Shi et al., 2011; Camara et al., 2015). Specifically, direct administration of anti-TNF-α medicine into the brain parenchyma or the cerebrospinal space for the treatment of HE has never been reported.

The aims of this study were two-fold. The first was to study the temporal profiles of TNF-α and ADMA in the plasma, cortex, and hippocampus of young BDL rats. The second aim was to examine whether peripherally with or without centrally administered etanercept was beneficial in preventing brain damage in young BDL rats, and if so, to examine the underlying mechanisms.

## Materials and Methods

### Animals and Experimental Design

All experiments were carried out according to the Guidelines for Animal Experiments of Chang Gung Memorial Hospital and Chang Gung University and approved by the Institutional Animal Care and Use Committee of the Kaohsiung Chang Gung Memorial Hospital (reference number: 2015031903). Male and female Sprague-Dawley rats were obtained (BioLASCO Taiwan, Taipei, Taiwan) for breeding. The day of delivery was defined as day 0. Male offspring rats at postnatal day (PND) 17 ± 1 weighing approximately 50 g were used. All animals were housed in a room maintained at 24°C with 12-h light/dark cycles. All animals had free access to standard chow and water.

**Figure 1:**
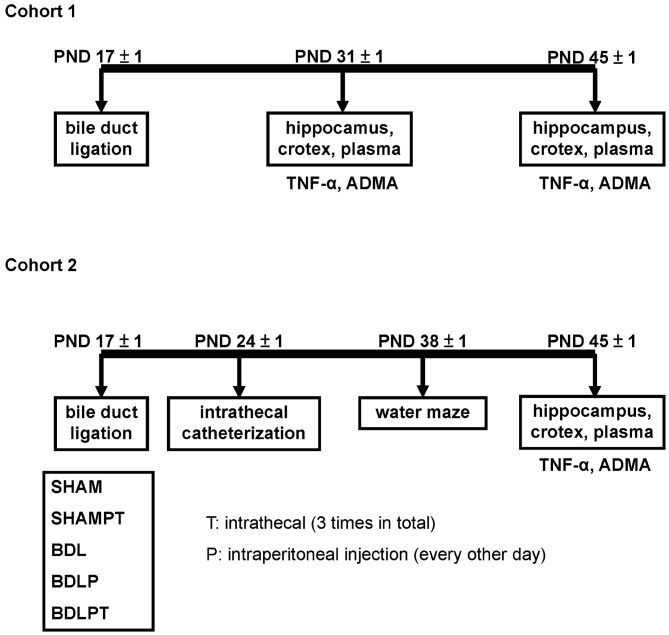
**The two subject cohorts used in this study.** Cohort 1 was used to compare the levels of tumor necrosis factor-α (TNF-α) and asymmetric dimethylarginine (ADMA) in the plasma, cortex, and hippocampus between the bile duct ligation (BDL) 2 week (BDL2W) or 4 week (BDL4W) group and the SHAM control group of rats. Cohort 2 was used to examine the changes in biochemistry, TNF-α, ADMA, and Morris water-maze performance in the BDL4W rats after etanercept treatment. SHAM, sham ligation for 4 weeks; SHAMPT, sham ligation for 4 weeks and injected with etanercept (10 mg/kg) intraperitoneally (IP) on postnatal day (PND) 17, 19, 21, 23, 25, 27, 29, 31, 33, 35, 37 and 39 (12 doses in total) and etanercept (100 μg) intrathecally (IT) on PND 31, 33, and 35 (3 doses in total); BDL, bile duct ligation for 4 weeks; BDLP, BDL plus etanercept IP, BDLPT, BDL plus etanercept IP and IT.

BDL rats were generated as we previously described (Sheen et al., 2010). We chose two time points, 2 weeks and 4 weeks, because 2 weeks of BDL represents the liver fibrogenesis stage and 4 weeks of BDL represents the chronic liver cirrhosis stage (Georgiev et al., 2008). In cohort 1, four experimental groups were defined. Rats that underwent sham treatment for 2 or 4 weeks at PND 17 ± 1 were designated as the SHAM2W group (*N* = 10) and the SHAM4W group (*N* = 10), respectively. Rats that underwent BDL for 2 or 4 weeks at PND 17 ± 1 were designated as the BDL2W group (*N* = 10) and BDL4W group (*N* = 10), respectively (Figure 1). In cohort 2, five experimental groups were defined. Rats that underwent sham ligation for 4 weeks at PND 17 ± 1 were designated as the SHAM group (*N* = 9). Rats that underwent sham ligation for 4 weeks at PND 17 ± 1 and were injected with etanercept (10 mg/kg) intraperitoneally (IP) every other day from PND 18 ± 1 to PND 38 ± 1 (12 doses in total) and etanercept (100 μg) intrathecally (IT) from PND 30 ± 1 to PND 38 ± 1 (3 doses in total) were designated as the SHAMPT group (*N* = 9). Rats that underwent BDL for 4 weeks at PND 17 ± 1 were designated as the BDL4W group (*N* = 9). Rats that underwent BDL for 4 weeks and were injected IP with etanercept were designated as the BDLP group (*N* = 9). Since BDL in rat is characterized by both peripheral and central inflammation (D’Mello et al., 2009), we therefore combined IT with IP etanercept to examine the possible effects. Rats that underwent BDL for 4 weeks and were injected IP and IT with etanercept were designated as the BDLPT group (*N* = 9; Figure 1). The doses of etanercept were chosen empirically, based on previous studies (Riazi et al., 2008; Chio et al., 2013; Camara et al., 2015).

### Measurement of Plasma Biochemistry Parameters

Blood samples were collected by cardiac puncture. Plasma was analyzed for aspartate aminotransferase (AST), alanine aminotransferase (ALT), ammonia, direct bilirubin and total bilirubin according to previously described methods (Huang et al., 2009).

### Liver and Brain Tissue Collection

Since we previously showed impairment of spatial learning and memory in young BDL rats (Huang et al., 2009), we examined two regions that are critically involved in spatial performance in rats: the cortex and hippocampus (Sloan et al., 2006; Spiers and Gilbert, 2015). Rats were euthanized on PND 45 with ketamine (50 mg/kg) and xylazine (50 mg/kg) and the cortices, hippocampi, and liver were removed immediately.

### Liver Pathology

Liver pathology was assessed on 4-μm section blocked in paraffin wax and stained with hematoxylin and eosin stain. The extent of liver injury was assessed on a blind basis using a histology activity index (HAI) score proposed by Knodell et al. (1981). Briefly, the HAI score examined liver cell necrosis and inflammation in four categories, including periportal necrosis, intralobular necrosis, portal inflammation, and fibrosis, with a total of 22 marks.

### Enzyme-Linked Immunosorbent Assay (ELISA)

Enzyme-linked immunosorbent assay (ELISA) analyses were performed using appropriate kits according to the manufacturer’s instructions, as previously reported (Huang et al., 2009). The parameters examined included plasma TNF-α, soluble TNF receptor 1 (sTNFR1), soluble TNF receptor 2 (sTNFR2), cortex TNF-α (R&D Systems, Inc, USA), plasma ADMA (Immunodiagnostic AG, Bensheim, Germany), and cortex and hippocampus ADMA (Bluegene, Shanghai, China).

### Western Blotting

Western blot (WB) analysis was performed on the cortex (100–200 μg total protein), as previously described (Tain et al., 2010a). We used 12% gels for separation of arginine methyltransferase 1 (PRMT1), dimethylarginine dimethyl-aminohydrolase 1 (DDAH1), DDAH2, and cationic amino acid transporter 1 (CAT1), and we used 8% gels for separation of endothelial nitric-oxide synthase (eNOS) and neuronal nitric-oxide synthase (nNOS). For TNF-α, we used a rabbit anti-TNF-α antibody (Millipore, Billerica, MA, USA, Cat# Ab1837P; 1:1000 dilution). For PRMT1, we used a rabbit anti-PRMT1 antibody (Millipore, Cat# 07-404; 1:2500 dilution). For DDAH, we used a goat anti-rat DDAH1 antibody (Santa Cruz, CA, USA, Cat# Sc26068 1:200 dilution) and a goat anti-rat DDAH2 antibody (Santa Cruz, Cat# Sc26071, 1:200 dilution). For CAT1, we used a rabbit anti-CAT1 antibody (Abcam, Cambridge, MA, USA; Cat# Ab37588; 1:250 dilution). For eNOS, we used a mouse anti-eNOS antibody (Transduction Laboratories, San Jose, CA, USA; Cat# 3340570; 1:250 dilution). For nNOS, we used a mouse anti-nNOS antibody (Santa Cruz, Cat# A11; 1:250 dilution), followed by a secondary donkey anti-goat antibody. Bands of interest were visualized using enhanced chemiluminescence (ECL) reagents (PerkinElmer, Waltham, MA, USA) and quantified by densitometry (Quantity One Analysis software; Bio-Rad, Hercules, CA, USA) as the integrated optical density after subtraction of background. The integrated optical density was normalized to Ponceau red staining (Ponceau S) to correct for any variations in total protein loading. The protein abundance was represented as integrated optical density/Ponceau S.

### Quantitative Real-Time PCR Analysis

PCR analysis was performed as reported previously (Tain et al., 2010b). In brief, RNA was extracted and reverse transcribed. Two-step quantitative real-time PCR was conducted using QuantiTect SYBR green PCR reagents (Qiagen, Valencia, CA, USA) according to the manufacturer’s protocol on a LightCycler 480 real-time PCR system (Roche Diagnostics Ltd., Taipei, Taiwan). β-Actin was used as a reference. The primers for TNF-α were 5′-GGCTGCCCCGACTACGT-3′ and 5′-AGGGCAAGGGCTCTTGATG-3′. The comparative threshold cycle (*C_T_*) method was employed for the relative quantification of gene expression. The averaged *C_T_* was subtracted from the corresponding averaged β-actin value for each sample (Δ*C_T_*). ΔΔ*C_T_* was obtained by subtracting the average control Δ*C_T_* value from the average experimental Δ*C_T_*. The fold-increase was established by calculating 2^−ΔΔ*CT*^ for experimental vs. control samples.

### Morris Water Maze: Spatial Memory

The Morris water-maze test was conducted to assess spatial learning and memory (Sheen et al., 2010) in all five groups of cohort 2 from PND 38 to PND 43. Briefly, at PND 38, each rat was habituated to the training environment. On PND 39, 40, 41 and 42, the rat was trained to find the submerged platform and this period was considered as the acquisition phase. At PND 43, retention of memory was tested with the platform absent.

### Astrocyte Cell Culture

Rat glioma C6 cell lines provided by American Type Culture Collection (ATCC) were grown in RPMI medium 1640 (Life Technologies) containing 4% fetal bovine serum and 8% horse serum at 37°C in a 5% CO_2_ incubator. Three groups were designated as follows: the first was control, the second was pre-treatment with rat TNF-α (10 pg/mL; R&D Systems) for 1 h, and the third was pre-treatment with rat TNF-α for 1 h with etanercept (0.1 μg/ml) added for 30 min. C6 cells were seeded (1 × 10^6^) onto glass slides overnight and fixed with 4% paraformaldehyde in phosphate-buffered saline (PBS) at 4°C for 15 min. The cells were then rinsed twice with PBS and permeabilized with 1% Triton X-100 for 7 min. Next, the cells were pretreated with 1% bovine serum albumin (BSA) in PBS at 25°C for 60 min, incubated with rabbit anti-ADMA polyclonal antibodies (Millipore) at a dilution of 1:1000 for 1 h, and treated with fluorescein isothiocyanate (FITC)-conjugated donkey anti-rabbit IgG polyclonal antibodies at a dilution of 1:10,000 for 1 h. Finally, the cells were washed with PBS, mounted in 90% glycerol containing 4′,6-diamidino-2-phenylindole (DAPI), and photographed under an immunofluorescent microscope (Olympus). In addition, the concentrations of ADMA in the whole cell lysate were examined by ELISA.

### Statistical Analysis

Biochemical parameters, liver pathology, cell culture results, WBs, PCR, ELISA, and retention in the Morris water maze were analyzed by one-way ANOVA with Tukey HSD *post hoc* test. For the Morris water maze spatial acquisition test, the average of the sum of the six trials per day was analyzed by ANOVA, considering group and day as independent variables, as well as by repeated-measure ANOVA. All analyses were performed using SPSS. Values were expressed as mean ± SEM and significance was defined as *P* < 0.05 for all tests.

## Results

### Cohort 1

**Figure 2:**
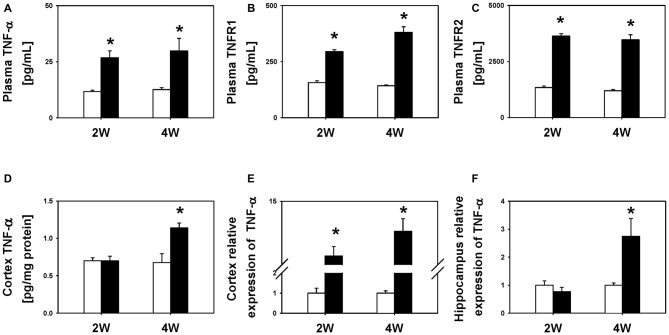
**TNF-α levels between BDL2W or BDL4W and SHAM control group rats.** Plasma TNF-α **(A)**, soluble tumor necrosis factor receptor 1 (sTNFR1) **(B)**, soluble tumor necrosis factor receptor 2 (sTNFR2) protein levels **(C)**, cortex TNF-α protein level **(D)**, cortex *TNF-α* mRNA **(E)** expression, and hippocampus *TNF-α* mRNA expression **(F)** in rats subjected to BDL for 2 weeks or 4 weeks, and the SHAM control. One-way ANOVA, followed by a Tukey HSD *post hoc* test was used to assess the statistical significance of differences between the SHAM and BDL groups, *P < 0.05.

To characterize the temporal profile of neuroinflammation following BDL, we compared the levels of TNF-α in the plasma and two regions of the brain, i.e., the cortex and hippocampus, between BDL and SHAM groups. We found that the BDL2W group rats had higher plasma TNF-α (26.8 ± 3.1 pg/mL vs. 11.7 ± 0.6 pg/mL, *P* = 0.031), sTNFR1 (294.8 ± 8.7 pg/mL vs. 156.2 ± 8.1 pg/mL, *P* < 0.001), and sTNFR2 (3632.5 ± 106.4 pg/mL vs. 1338.5 ± 65.8 pg/mL, *P* < 0.001) levels as well as higher cortex *TNF-α* mRNA expression (4.67 ± 1.76 vs. 1.00 ± 0.24, *P* < 0.001) than the SHAM controls (Figure 2), but there were no significant differences in cortex TNF-α protein levels (0.70 ± 0.06 pg/mg protein vs. 0.70 ± 0.04 pg/mg protein, *P* = 1.00) or hippocampus *TNF-α* mRNA expression (0.77 ± 0.15 vs. 1.00 ± 0.15, *P* = 0.660). Interestingly, the BDL4W group rats had significantly higher cortex TNF-α protein levels (1.14 ± 0.06 pg/mg protein vs. 0.71 ± 0.13 pg/mg protein, *P* = 0.030), and hippocampus *TNF-α* mRNA expression (2.74 ± 0.64 vs. 1.00 ± 0.08, *P* = 0.002), as well as higher plasma TNF-α (29.8 ± 5.6 pg/mL vs. 12.6 ± 0.8 pg/mL, *P* = 0.004), sTNFR1 (380.1 ± 25.5 pg/mL vs. 142.5 ± 4.0 pg/mL, *P* < 0.001), and sTNFR2 (3468.6 ± 225.6 pg/mL vs. 1196.1 ± 51.9 pg/mL, *P* < 0.001) levels and higher cortex *TNF-α* mRNA expression (9.34 ± 2.36 vs. 1.00 ± 0.11, *P* < 0.001) than the SHAM controls (Figure 2).

**Figure 3:**
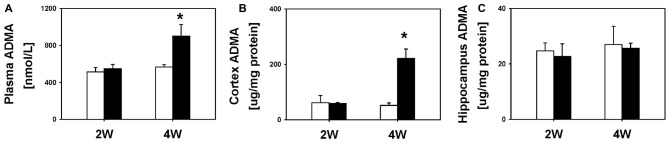
**ADMA levels in BDL 2W or BDW 4W and SHAM control rats.** Plasma **(A)**, cortex **(B)** and hippocampus **(C)**. One-way ANOVA, followed by a Tukey HSD *post hoc* test, was used to assess the statistical significance of differences between SHAM and BDL groups, *P < 0.05.

Next, we examined the temporal profile of ADMA after BDL. We did not find significant differences in plasma (549.0 ± 44.3 nmol/L vs. 511.8 ± 48.1 nmol/L), cortex (58.9 ± 4.0 μg/mg protein vs. 61.9 ± 26.0 μg/mg protein) or hippocampus ADMA levels (22.7 ± 4.6 μg/mg protein vs. 24.7 ± 2.9 μg/mg protein; all *P* = 1.00) between the BDL2W group and SHAM controls (Figure 3). However, the BDL4W group rats had higher plasma (899.9 ± 128.6 nmol/L vs. 564.8 ± 26.4 nmol/L, *P* = 0.02) and cortex ADMA levels (221.9 ± 33.1 μg/mg protein vs. 52.2 ± 9.3 μg/mg protein, *P* = 0.003), while there was no significant difference in the hippocampus levels (25.6 ± 1.9 μg/mg protein vs. 27.0 ± 6.6 μg/mg protein, *P* = 1.00) as compared with the SHAM controls (Figure 3). Overall, the BDL4W group rats had higher plasma and cortex TNF-α and ADMA levels than the SHAM controls. Therefore, in the subsequent cohort 2 study, we used BDL4W group rats as a model for testing the response to etanercept.

### Cohort 2

Given that etanercept does not cross the BBB, we treated BDL4W rats with etanercept IP with/without IT etanercept administration, to test the effect of anti-TNF-α therapy.

### Liver Transaminase and Pathology

**Figure 4:**
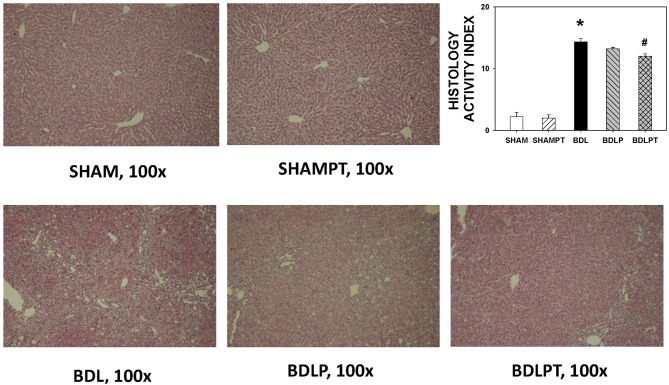
**Liver injury assessed by hematoxylin and eosin staining using a histology activity index (HAI) score.** One-way ANOVA, followed by a Tukey HSD *post hoc* test, was used to assess the statistical significance of differences among groups, *P < 0.05 vs. SHAM; ^#^*P* < 0.05 vs. BDL4W.

BDL4W group rats had higher plasma direct and total bilirubin, AST, ALT, and ammonia levels than the SHAM group (Table 1). The increases in AST, ALT, and ammonia levels were not reversed in the BDLP or BDLPT group. Rats from the BDL4W group had higher HAI scores compared to the SHAM group (14.33 ± 0.53 vs. 2.20 ± 0.73, *P* < 0.001; Figure 4). The BDLPT group had lower HAI scores compared to the BDL4W group (12.00 ± 0.38 vs. 14.33 ± 0.53, *P* < 0.001; Figure 4). In parallel, the BDLPT group had lower direct/total bilirubin compared to the BDL4W group (Table 1).

**Table 1:** **Clinical parameters**.

	SHAM *N* = 9	SHAMPT *N* = 9	BDL *N* = 9	BDLP *N* = 9	BDLPT *N* = 9
AST (IU/L)	91.1 ± 4.7	99.9 ± 6.0	359.1 ± 48.5*	377.1 ± 50.1	396.7 ± 30.9
ALT (IU/L)	48.7 ± 1.8	45.4 ± 2.9	128.7 ± 18.8*	125.1 ± 13.1	140.6 ± 8.8
Direct bilirubin (mg/dL)	0.1 ± 0.0	0.1 ± 0.0	4.4 ± 0.3*	4.6 ± 0.2	3.5 ± 0.1^#^
Total bilirubin (mg/dL)	0.4 ± 0.0	0.3 ± 0.0	6.4 ± 0.5*	6.7 ± 0.5	4.9 ± 0.2^#^
Ammonia (μg/dL)	112.4 ± 10.9	96.6 ± 7.2	461.4 ± 57.4*	479.6 ± 63.2	468.6 ± 61.4
Body weight (g)	246.4 ± 5.3	212 ± 6.2	205.6 ± 9.7*	175.3 ± 8.9^#^	189.7 ± 3.8

Values are means ± SEM; AST, aspartate aminotransferase; ALT, alanine aminotransferase; SHAM, sham ligation for 4 weeks; SHAMPT, SHAM and etanercept intraperitoneally (IP) plus intrathecally (IT); BDL, bile duct ligation for 4 weeks; BDLP, BDL plus etanercept IP; BDLPT, BDL plus etanercept IP and IT, *P < 0.05 vs. SHAM; ^#^P < 0.05 vs. BDL.

### Plasma and Brain TNF-α and ADMA

**Figure 5:**
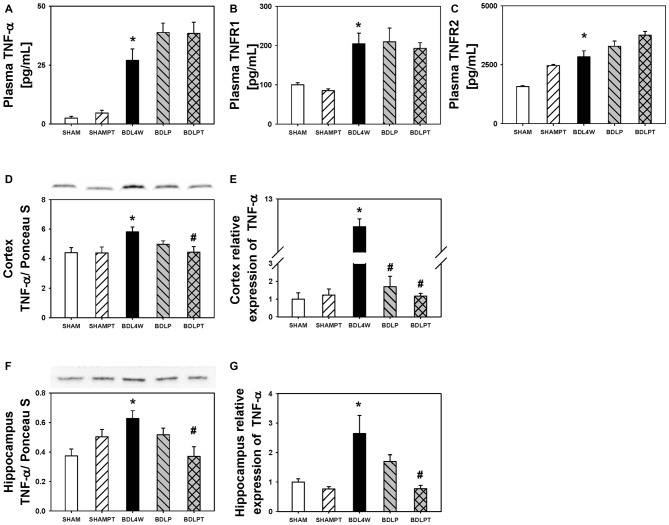
**Changes in TNF-α after etanercept treatment.** Plasma protein levels of TNF-α **(A)**, sTNFR1 **(B)**, sTNFR2 **(C)**, levels of cortex TNF-α protein **(D)**, and mRNA **(E)** expression, and hippocampus TNF-α protein **(F)** and mRNA **(G)** expressions in the five groups. SHAM, sham ligation for 4 weeks; SHAMPT, SHAM and etanercept IP plus IT; BDL, bile duct ligation for 4 weeks; BDLP, BDL plus etanercept IP, BDLPT, BDL plus etanercept IP and IT. One-way ANOVA, followed by a Tukey HSD *post hoc* test, was used to assess the statistical significance of differences among groups, *P < 0.05 vs. SHAM; ^#^*P* < 0.05 vs. BDL4W.

Cortex TNF-α protein levels were increased in the BDL4W group compared to SHAM controls (*P* = 0.037) and this increase was reduced in the BDLPT group only (*P* = 0.042). *TNF-α* mRNA expression in the cortex was increased in the BDL4W group compared with SHAM controls (8.29 ± 1.35 vs. 1.00 ± 0.35, *P* < 0.001), and this increase was reduced in both the BDLP and BDLPT groups (cortex BDL4W vs. BDLP 8.29 ± 1.35 vs. 1.70 ± 0.59, *P* < 0.001; BDL4W vs. BDLPT 8.29 ± 1.35 vs. 1.17 ± 0.05, *P* < 0.001). *TNF-α* mRNA expression and protein levels in the hippocampus were both increased in the BDL4W group compared with SHAM controls (*P* = 0.001; *P* = 0.010), and these increases were both reduced only in the BDLPT groups (*P* = 0.008; *P* = 0.009; Figure 4). The plasma levels of TNF-α, sTNFR1, and sTNFR2 were increased in the BDL4W group compared with SHAM controls (27.0 ± 4.9 pg/mL vs. 2.5 ± 0.7 pg/mL, *P* < 0.001; 204.4 ± 27.1 pg/mL vs. 100.1 ± 4.9 pg/mL, *P* = 0.012; 2835.8 ± 25.3 pg/mL vs. 1574.1 ± 45.3 pg/mL, *P* < 0.001); however, these were not reduced in either the BDLP or BDLPT group (Figure 5).

**Figure 6:**
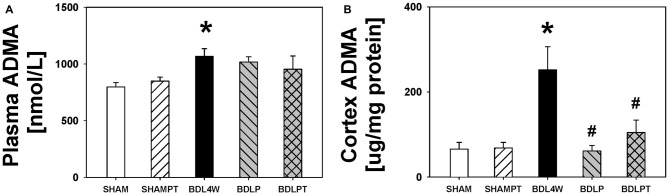
**ADMA changes after etanercept treatment.** Plasma ADMA **(A)** and cortex ADMA **(B)** in the five groups. SHAM, sham ligation for 4 weeks; SHAMPT, SHAM and etanercept IP plus IT; BDL, bile duct ligation for 4 weeks; BDLP, BDL plus etanercept IP, BDLPT, BDL plus etanercept IP and IT. One-way ANOVA, followed by a Tukey HSD *post hoc* test, was used to assess the statistical significance of differences among groups, *P < 0.05 vs. SHAM; ^#^*P* < 0.05 vs. BDL4W.

The level of ADMA in the cortex was increased in the BDL4W group compared with SHAM controls (251.9 ± 54.4 μg/mg protein vs. 61.0 ± 13.2 μg/mg protein, *P* = 0.001) and this increase was reduced in both the BDLP and BDLPT groups (251.9 ± 54.4 μg/mg protein vs. 65.8 ± 15.6 μg/mg protein, *P* = 0.001; 251.9 ± 54.4 vs. 104.5 ± 29.2, *P* = 0.009). The plasma level of ADMA was increased in the BDL4W group compared with SHAM controls (1067.3 ± 67.7 nmol/L vs. 797.7 ± 38.4 nmol/L, *P* = 0.014), yet this increase was not reduced in either the BDLP or BDLPT group (1067.3 ± 67.7 nmol/L vs. 1016.8 ± 46.1 nmol/L, *P* = 0.971; 1067.3 ± 67.7 nmol/L vs. 954.1 ± 117.1 nmol/L, *P* = 0.716; Figure 6). These data showed that etanercept treatment could reduce cortex and/or hippocampus TNF-α and ADMA levels but had no marked effect on plasma TNF-α or ADMA in BDL rats. Table 2 is a summary of TNF-α and ADMA changes following peripheral and/or central etanercept administration.

**Table 2:** **Effects of etanercept on increased peripheral and central TNF-α and Asymmetric dimethylarginine (ADMA) in bile duct ligation (BDL) rats**.

	TNF-α				ADMA	
Group	Cortex protein	Cortex mRNA	Hippocampus protein	Hippocampus mRNA	Plasma	Cortex		
BDLP	–	↓	–	–	–	↓
BDLPT	↓	↓	↓	↓	–	↓

*BDLP, Bile duct ligation (BDL) plus etanercept intraperitoneally (IP); BDLPT, BDL plus etanercept IP and intrathecally; –, no significant changes as compared with BDL group; ↓, decreased significantly as compared with BDL group*.

**Figure 7:**
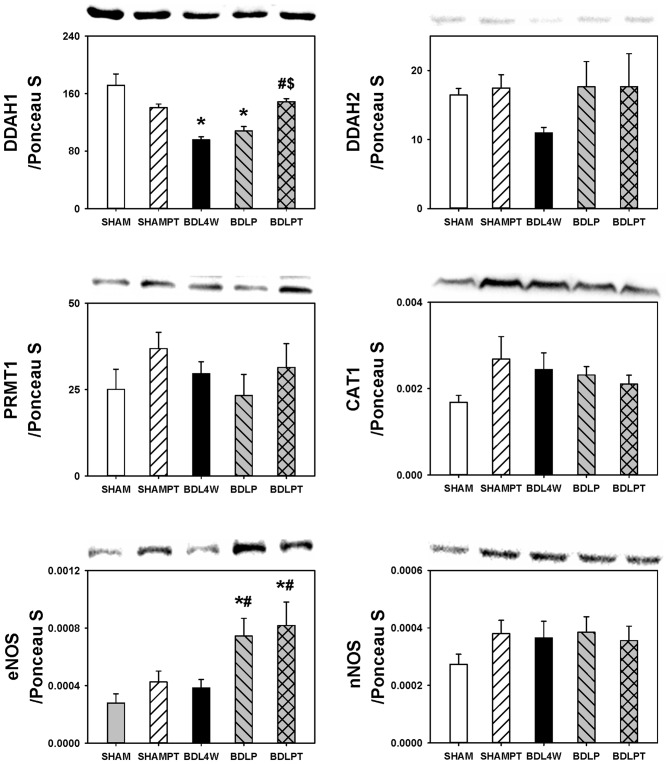
**Changes in expression of molecules related to ADMA metabolism after etanercept treatment.** DDAH1, Dimethylarginine dimethyl-aminohydrolase 1; DDAH2, dimethylarginine dimethyl-aminohydrolase 2; PRMT 1, protein arginine methyltransferase 1; CAT 1, cationic amino acid transporter ; eNOS, endothelial nitric-oxide synthase, nNOS, neuronal nitric-oxide synthase; SHAM, sham ligation for 4 weeks; SHAMPT, SHAM and etanercept IP plus IT; BDL, bile duct ligation for 4 weeks; BDLP, BDL plus etanercept IP, BDLPT, BDL plus etanercept IP and IT. One-way ANOVA followed by Tukey HSD *post hoc* test was used to assess the statistically significances among groups, *P < 0.05 vs. SHAM; ^#^*P* < 0.05 vs. BDL4W; ^$^*P* < 0.05 vs. BDLP.

In order to investigate whether the normalization of cortex TNF-α and ADMA in BDL rats after etanercept treatment was related to the metabolism of ADMA, we examined the cortex expression of DDAH1, DDAH2, PRMT1, CAT1, eNOS, and nNOS. The level of DDAH1 was decreased in the cortex in the BDL4W group compared with SHAM controls and this decrease of DDAH1 was rescued in the BDLPT group only (Figure 7). No significant differences were found in the cortex levels of DDAH2, PRMT1, CAT1, eNOS, or nNOS between SHAM and BDL4W groups (Figure 7).

### Morris Water Maze: Spatial Memory

**Figure 8:**
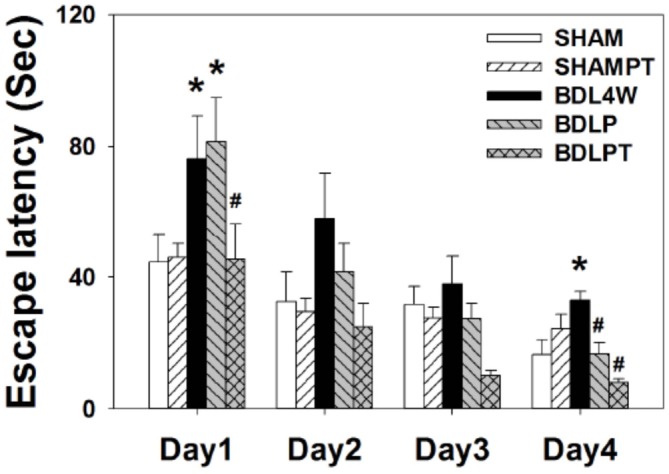
**Latencies of escape to the platform in the Morris water maze (mean ± SEM).** Rats in the BDL4W group swam for a longer time while seeking to find the submerged platform on days 1 and 4 of acquisition than did the SHAM group rats. Combined intraperitoneal and intrathecal etanercept shortened the swim latency to a level comparable to the SHAM group on all 4 days of acquisition. However, etanercept IP had no significant effect on swim latency. Two-way ANOVA with repeated measures was used to assess the statistical significance of differences among groups, *P < 0.05 vs. SHAM; ^#^*P* < 0.05 vs. BDL4W.

Given that etanercept treatment normalized cortex and/or hippocampus TNF-α and ADMA in BDL rats, we then investigated whether this treatment could mitigate spatial impairment in HE rats. Figure 8 showed the mean latencies of escape to the hidden platform as a function of days.

Acquisition: the water maze tests revealed that all rats were able to learn how to find the platform and that there was no significant difference in swim velocity between the different treatment groups at any time (*P* > 0.1). Two-factor ANOVA revealed significant differences among the groups for the number of trial blocks needed to learn to escape by swimming with visual cues (*F*_(4,30)_ = 3.31, *P* = 0.023). Escape latencies improved over time in all five groups as indicated by a significant effect of day (*F*_(3,120)_ = 24.24, *P* < 0.001), indicating that learning occurred (Figure 8). An interaction between group and days required to learn the task was also present (days: treatment groups, *F* = 3.99, *P* < 0.05).

There were group differences on day 1 (*F*_(4,30)_ = 3.73, *P* = 0.014) and 4 (*F*_(4,30)_ = 3.61, *P* = 0.016) of acquisition. A *post hoc* test showed that BDLPT rats performed significantly better than BDL4W and BDLP rats on day 1 (*P* < 0.05) and as well as SHAM and SHAMPT rats (*P* > 0.05). On day 4, BDLPT rats performed significantly better than BDL4W and BDLP rats (*P* < 0.05) and as well as SHAM and SHAMPT rats (*P* > 0.05). Dually IP/IT etanercept administration ameliorated the spatial deficit, as rats in this group demonstrated a similar performance to SHAM group rats and had faster acquisition as compared with BDL4W group rats (*P* = 0.004). However, IP etanercept had no significant effect on the spatial deficit.

Retention: the “free swim” trial performed immediately after the last trial on day 5 revealed no significantly retention differences among the five groups (*F*_(4,31)_ = 0.601; *P* = 0.665).

### Astrocyte Cell Culture and Immunofluorescence

**Figure 9:**
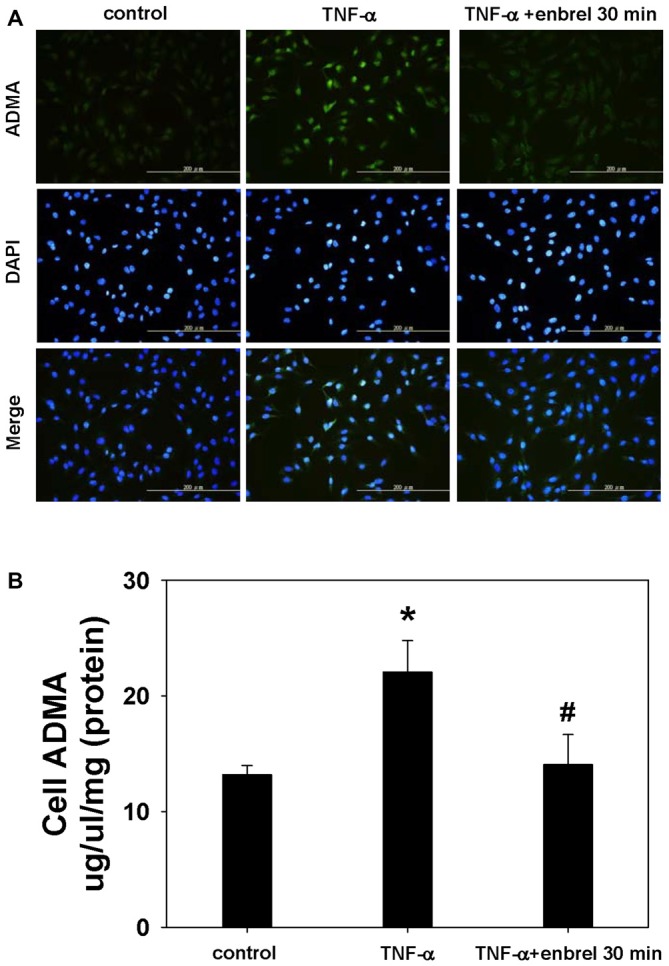
**Staining of ADMA in intact cells and visualization by fluorescence microscopy. (A)** Representative images acquired from cultures of rat astrocyte C6 cell lines. ADMA staining is shown in the upper panel. DNA was stained with 4′,6-diamidino-2-phenylindole (DAPI; middle panels). Merged images are shown in the lower panels. **(B)** The concentration of ADMA in whole cell lysates. One-way ANOVA followed by Tukey HSD *post hoc* test was used to assess the statistically significances among groups, *P < 0.05 vs. control; ^#^*P* < 0.05 vs. TNF-α incubated only.

To determine whether etanercept can decrease the TNF-α induced ADMA production, we treated the astrocyte cell line C6 with TNF-α and applied etanercept for 30 min. We found that immunofluorescence staining intensity of ADMA was increased after TNF-α exposure and decreased after etanercept application. In addition, the concentration of ADMA in the whole cell lysate was increased following exposure to TNF-α (22.0 ± 2.8 nmol/L vs. 13.1 ± 0.8 nmol/L, *P* = 0.014) and etanercept blocked this increase in ADMA (14.0 ± 2.6 nmol/L vs. 22.0 ± 2.8 nmol/L, *P* = 0.025; Figure 9).

## Discussion

The main findings of this study were as follows: (1) the BDL4W group of young rats had higher plasma TNF-α, sTNFR1, sTNFR2, ADMA levels, higher hippocampus TNF-α mRNA expressions and protein levels, and cortex ADMA, TNF-α mRNA expressions and protein levels than SHAM controls; (2) the increased cortex *TNF-α* mRNA expression and cortex ADMA concentrations in BDL4W rats were reduced in both the BDLP and BDLPT groups, while increased cortex TNF-α protein and hippocampus TNF-α mRNA and protein expression in BDL4W rats were reduced in the BDLPT group alone; and (3) the BDL4W group of young rats exhibited spatial deficits as compared to SHAM and SHAMPT group rats, and combined IP and IT etanercept administration (BDLPT) rescued these effects.

In the central nervous system, microglia and astrocytes are believed to be the primary sources of TNF-α; TNF-α (through activation of TNFR1) disrupts learning and memory and regulates neuronal death. TNF-α has been implicated in the pathogenesis of a wide number of neurological disorders that develop both acutely, as in traumatic brain injury and stroke, and chronically, as in Alzheimer’s disease and Parkinson’s disease (McCoy and Tansey, 2008; Tobinick and Gross, 2008; Tuttolomondo et al., 2008).

Previous studies have proposed serum sTNFR levels to be markers of TNF system activation (Spengler et al., 1996; Naveau et al., 1998). Serum levels of TNF-α, sTNFR1, and sTNFR2 were also reported to be higher in cirrhotic patients than those in the controls and were correlated with the disease severity (Shiraki et al., 2010). In addition, higher serum sTNFR2 concentrations were also related to higher mortality in cirrhotic patients (Grünhage et al., 2008). Here we found higher plasma TNF-α, sTNFR1, and sTNFR2 levels in both the BDL 2W and BDL 4W group rats than their relative SHAM controls. Our finding suggested alterations of peripheral TNF also contributed to the pathogenesis of BDL-induced HE.

There are many mechanisms involved in bi-directional liver-brain proinflammatory signaling (D’Mello et al., 2013, 2015; D’Mello and Swain, 2014). Several studies have shown that TNF-α-secreting monocytes are recruited into the brain in BDL rats and mice (Jiang et al., 2008; D’Mello and Swain, 2014). In the Morris water-maze task, rats subjected to BDL showed a progressive impairment of spatial memory from 7 to 28 days after the BDL procedure (Dhanda and Sandhir, 2015). In this study, we found that there were higher plasma TNF-α levels in the BDW 2W and BDW 4W group rats than their relative SHAM controls. However, cortical TNF-α protein was increased only in the BDL4W group rat. These findings suggested that there was a discrepancy between brain and peripheral systems in terms of temporal progression of TNF-α.

Systemic administration of anti-TNF-α therapy attenuates increased brain levels of TNF-α in brain disorders (Chio et al., 2013; Detrait et al., 2014). Other studies also showed improved brain functional outcomes after systemically administered anti-TNF-α therapy (Chio et al., 2013; Detrait et al., 2014; Jiang et al., 2008; Clausen et al., 2014). Deletion of TNF receptor genes delays the onset of HE and attenuates brain edema in azoxymethane-induced acute liver failure (Bémeur et al., 2010). The present study demonstrated that IP etanercept administration lowered the increased *TNF-α* mRNA expression in the cortex, while dually IP/IT etanercept administration lowered increased cortex and hippocampus TNF-α mRNA and protein expressions, which was accompanied by improved spatial learning. Our data suggests that increased cortex and hippocampus TNF-α levels may play a role in spatial deficits in BDL-induced HE.

Both young and adult BDL rats have spatial memory deficits (Sheen et al., 2010; Javadi-Paydar et al., 2013). Although the correspondence between developmental stages across the two species is not exact, PND 17 in rats can be considered as corresponding to early childhood in humans (Clancy et al., 2001; Avishai-Eliner et al., 2002). The timing of insults in the developing CNS is pivotal to the outcome, because the development and maturation of synapses (Anderson et al., 2011), cytokine, chemokine (Schoderboeck et al., 2009), and neurotransmitter expression (Herlenius and Lagercrantz, 2001), BBB maturation (Ballabh et al., 2004), redox milieus (Miller et al., 2012), and oscillation networks (Tau and Peterson, 2010) vary widely during development. All of these parameters are already known to be important pathogenetic factors in HE (Celik et al., 2005; Häussinger and Schliess, 2008; Rodrigo et al., 2010; Dhanda and Sandhir, 2015). Therefore, it is important to study young animals with HE for both experimental and clinical reasons.

Few studies have used the intrathecal/intraventricular route to deliver anti-TNF-α drugs to treat brain disease (Heldmann et al., 2005; Riazi et al., 2008; Medeiros et al., 2010; Shi et al., 2011; Camara et al., 2015). Intraventricular etanercept administration prevents both synaptic loss and disruption of memory consolidation in a mouse model of Alzheimer’s disease (Medeiros et al., 2010). Direct intrathecal/intraventricular administration of substances is markedly more effective for inducing behavioral changes than the peripheral route (Adam et al., 2006; Veening and Barendregt, 2010). BDL in rats is characterized by both peripheral and central inflammation (D’Mello et al., 2009). In this study, we found that the dual IP/IT administration of etanercept was effective to rescue spatial deficits in young rats with BDL. The beneficial effects of IT administration can be due to some physicochemical factors. The cerebrospinal fluid may act as a broadcasting system for coordinated delivery of messages to a variety of nearby and distant brain areas. Some form of volume transmission may also underlie the changes in the outcome. However, we could not exclude the possibility that IT etanercept alone is effective in reducing peripheral and central inflammation in BDL rats found in this study. In clinical practice, severe liver disease may complicate with hepatopulmonary syndrome, hepatorenal syndrome, HE etc., and the treatment include correction of the underlying disease and management of the various complications (Møller and Bendtsen, 2015). While in the brain, the therapy of HE is aimed at the treatment and prevention of the disease-precipitating factors, including infections, electrolyte disturbances, bleeding, and elevated ammonia levels (Häussinger and Sies, 2013). BDL in rats is characterized by both peripheral and central inflammation. Peripheral monocytes are recruited into the brain and initiated microglia activation and neuroinflammation (D’Mello and Swain, 2014). Considering the low benefit to risk ratio, this pre-clinical study did not investigate the effects of IT-administered etanercept alone in BDL rats. The role of IT-administered etanercept alone and its contribution to the combined therapy in BDL rats with HE require further investigation.

The glutamate-NO-cyclic guanosine monophosphate (cGMP) pathway is impaired in the brain of chronic moderate hyperammonemia and HE (Corbalán et al., 2002). NO, which is inhibited by ADMA, is critically involved in spatial memory function (de la Torre and Aliev, 2005). Balasubramaniyan et al. (2012) demonstrated that brain ADMA levels were significantly higher in adult rats 4 weeks after BDL and these ADMA levels were reduced after treatment with ornithine phenylacetate. They also showed a concomitant increase in brain TNF-α level. Likewise, Bajaj et al. (2013) found that patients with liver cirrhosis had poor cognition and higher serum ADMA levels. Both TNF-α and ADMA were increased in plasma and the brain in encephalopathy induced by acute (Milewski et al., 2015) or chronic liver failure (Balasubramaniyan et al., 2012). In this study, we treated the astrocyte cell line C6 with TNF-α and found that ADMA was increased in whole cell lysates following exposure to TNF-α and etanercept blocked this increase in ADMA. We also demonstrated that young rats with BDL for 4 weeks had increased cortex ADMA levels and that the cortical DDAH1 levels were downregulated in BDL rats and upregulated after dual IP/IT etanercept treatment.

In this study, dually IP/IT etanercept injections reduced the TNF-α mRNA and protein levels in both the cortex and the hippocampus. However, IP-administered etanercept reversed the BDL-induced increases only in the cortical TNF-α mRNA and ADMA protein levels. It affected neither the cortical TNF-α protein expression nor the hippocampal TNF-α mRNA and protein levels. The discordant response of TNF-α mRNA and protein expression to the treatment may be related to differences in mRNA export, ribosome recruitment to mRNA affecting translation efficiency, protein stability, or microRNAs (Carpenter et al., 2014; Vélez-Bermúdez and Schmidt, 2014). Combined with our water maze testing data, these results suggest that changes in cortical and hippocampal TNF-α expression might play a major role in rescuing the spatial deficit in young BDL rats. Cortical ADMA expression appears to play only a minor role in this process. However, the role of increased ADMA expression in cognition needs further investigation.

It is intriguing that the levels of both ADMA and TNF-α were more readily decreased in the brain than the plasma by peripheral or central etanercept administration. There are several possibilities. First, we measured plasma TNF-α by ELISA and cortex TNF-α by WB through recognizing the epitope in the cohort 2 study. TNF bound to etanercept can still be present and detectable but be unable to mediate biological or inflammatory effects (Bhatia and Kast, 2007). Different detection tools that recognize dissimilar epitope may result in the inconsistent responses between plasma and brain. Next, the liver and brain was interacted in inflammatory liver disease. In the context of BDL, the production of peripheral inflammatory signals may affect the brain through humoral or neural routes as well as through active recruitment of monocytes into the brain (D’Mello et al., 2009; Butterworth, 2013; D’Mello and Swain, 2014). The afferent and efferent components of the vagus nerve play a role in controlling both systemic and central inflammation. Vagal afferents can directly sense cytokine levels in the blood via specific cytokine receptors that are present on the endings of these nerves in the paraganglia located in the gastrointestinal tract, spleen, bone marrow, liver, and heart. These nerves transmit the signals to other brain regions to modulate inflammation (Goehler et al., 2000; Tracey, 2002). Vagal nerve may sense the systemic inflammation and reflex release of acetylcholine in organs to reduce systemic inflammation by reducing proinflammatory cytokines (Borovikova et al., 2000). Therefore, IP-administered etanercept did not reduce the TNF-α and ADMA levels in the plasma significantly, yet it might affect the routes connected with the brain and decrease brain inflammation to a significant extent. On the other hand, central signaling may also convey the information to the liver through the sympathetic and parasympathetic branches of the vagus nerve (Montiel-Castro et al., 2013; Rosas-Ballina et al., 2015; Tanida et al., 2015), so that dual IT/IP may ameliorate liver histopathology and normalize direct and total bilirubin levels. The mechanisms of the peripheral and central production and elimination of inflammatory molecules are very complex and varied (D’Mello et al., 2009, 2015; Teerlink et al., 2009; Sheen et al., 2014).

## Conclusions

HE is a frequent and serious complication of chronic liver failure. This study demonstrated that young BDL rats exhibited increased circulating and brain TNF-α and ADMA levels, as well as spatial deficits, as in adult BDL rats, indicating that increases in and interaction between TNF-α and ADMA play an important role in HE. Moreover, combined IP and IT etanercept reduce spatial deficits in young BDL rats. Appropriate targeting of TNF-α and ADMA may offer a new treatment avenue for HE.

## Author Contributions

J-MS: performed the majority of the experiments and wrote this manuscript. Y-CC: aided in experimental design and behavioral testing. M-HH: assisted with experimental design and aided in serum analysis. Y-LT and H-RY: assisted with planning and interpreting behavioral testing. L-TH: designed the study, edited and provided final approval of this manuscript. All authors read and approved the final manuscript.

## Conflict of Interest Statement

The authors declare that the research was conducted in the absence of any commercial or financial relationships that could be construed as a potential conflict of interest.

## References

[B1] Adam C. L. Findlay P. A. Miller D. W. (2006). Blood-brain leptin transport and appetite and reproductive neuroendocrine responses to intracerebroventricular leptin injection in sheep: influence of photoperiod. Endocrinology 147, 4589–4598. 10.1210/en.2006-0576 16794008

[B2] Anderson V. Spencer-Smith M. Wood A. (2011). Do children really recover better? Neurobehavioural plasticity after early brain insult. Brain 134, 2197–2221. 10.1093/brain/awr103 21784775

[B3] Angel K. Provan S. A. Mowinckel P. Seljeflot I. Kvien T. K. Atar D. (2012). The L-arginine/asymmetric dimethylarginine ratio is improved by anti-tumor necrosis factor-α therapy in inflammatory arthropathies. Associations with aortic stiffness. Atherosclerosis 225, 160–165. 10.1016/j.atherosclerosis.2012.08.033 23014354

[B4] Avishai-Eliner S. Brunson K. L. Sandman C. A. Baram T. Z. (2002). Stressed-out, or in (utero)? Trends Neurosci. 25, 518–524. 10.1016/s0166-2236(02)02241-5 12220880 PMC2930786

[B5] Bajaj J. S. Ahluwalia V. Wade J. B. Sanyal A. J. White M. B. Noble N. A.. (2013). Asymmetric dimethylarginine is strongly associated with cognitive dysfunction and brain MR spectroscopic abnormalities in cirrhosis. J. Hepatol. 58, 38–44. 10.1016/j.jhep.2012.08.005 22889958 PMC3508094

[B6] Balasubramaniyan V. Wright G. Sharma V. Davies N. A. Sharifi Y. Habtesion A.. (2012). Ammonia reduction with ornithine phenylacetate restores brain eNOS activity via the DDAH-ADMA pathway in bile duct-ligated cirrhotic rats. Am. J. Physiol. Gastrointest. Liver Physiol. 302, G145–G152. 10.1152/ajpgi.00097.2011 21903766

[B7] Ballabh P. Braun A. Nedergaard M. (2004). The blood-brain barrier: an overview: structure, regulation and clinical implications. Neurobiol. Dis. 16, 1–13. 10.1016/j.nbd.2003.12.016 15207256

[B8] Banks W. A. Plotkin S. R. Kastin A. J. (1995). Permeability of the blood-brain barrier to soluble cytokine receptors. Neuroimmunomodulation 2, 161–165. 10.1159/000096887 8646566

[B9] Bémeur C. Qu H. Desjardins P. Butterworth R. F. (2010). IL-1 or TNF receptor gene deletion delays onset of encephalopathy and attenuates brain edema in experimental acute liver failure. Neurochem. Int. 56, 213–215. 10.1016/j.neuint.2009.11.010 19931338

[B10] Bhatia A. Kast R. E. (2007). Tumor necrosis factor (TNF) can paradoxically increase on etanercept treatment, occasionally contributing to TNF-mediated disease. J. Rheumatol. 34, 447–449. 17304667

[B11] Borovikova L. V. Ivanova S. Zhang M. Yang H. Botchkina G. I. Watkins L. R.. (2000). Vagus nerve stimulation attenuates the systemic inflammatory response to endotoxin. Nature 405, 458–462. 10.1038/35013070 10839541

[B12] Butterworth R. F. (2013). The liver-brain axis in liver failure: neuroinflammation and encephalopathy. Nat. Rev. Gastroenterol. Hepatol. 10, 522–528. 10.1038/nrgastro.2013.99 23817325

[B13] Camara M. L. Corrigan F. Jaehne E. J. Jawahar M. C. Anscomb H. Baune B. T. (2015). Effects of centrally administered etanercept on behavior, microglia and astrocytes in mice following a peripheral immune challenge. Neuropsychopharmacology 40, 502–512. 10.1038/npp.2014.199 25103178 PMC4443965

[B14] Carpenter S. Ricci E. P. Mercier B. C. Moore M. J. Fitzgerald K. A. (2014). Post-transcriptional regulation of gene expression in innate immunity. Nat. Rev. Immunol. 14, 361–376. 10.1038/nri3682 24854588

[B15] Celik T. Gören M. Z. Cinar K. Gürdal H. Onder F. O. Tan A.. (2005). Fatigue of cholestasis and the serotoninergic neurotransmitter system in the rat. Hepatology 41, 731–737. 10.1002/hep.20617 15726642

[B16] Chastre A. Bélanger M. Beauchesne E. Nguyen B. N. Desjardins P. Butterworth R. F. (2012). Inflammatory cascades driven by tumor necrosis factor-alpha play a major role in the progression of acute liver failure and its neurological complications. PLoS One 7:e49670. 10.1371/journal.pone.0049670 23166746 PMC3499491

[B19] Chen X. M. Xia J. Zhou T. Yuan Q. Zhang W. F. Hu C. P.. (2013). Involvement of DDAH/ADMA pathway in the pathogenesis of rheumatoid arthritis in rats. Int. Immunopharmacol. 16, 322–331. 10.1016/j.intimp.2013.04.009 23619555

[B18] Chen M. F. Xie X. M. Yang T. L. Wang Y. J. Zhang X. H. Luo B. L.. (2007). Role of asymmetric dimethylarginine in inflammatory reactions by angiotensin II. J. Vasc. Res. 44, 391–402. 10.1159/000103284 17551258

[B17] Chen M. Zhang L. (2011). Epigenetic mechanisms in developmental programming of adult disease. Drug Discov. Today 16, 1007–1018. 10.1016/j.drudis.2011.09.008 21945859 PMC3226870

[B20] Chio C. C. Chang C. H. Wang C. C. Cheong C. U. Chao C. M. Cheng B. C.. (2013). Etanercept attenuates traumatic brain injury in rats by reducing early microglial expression of tumor necrosis factor-α. BMC. Neurosci. 14:33. 10.1186/1471-2202-14-33 23496862 PMC3636122

[B21] Clancy B. Darlington R. B. Finlay B. L. (2001). Translating developmental time across mammalian species. Neuroscience 105, 7–17. 10.1016/s0306-4522(01)00171-3 11483296

[B22] Clark I. A. Alleva L. M. Vissel B. (2010). The roles of TNF in brain dysfunction and disease. Pharmacol. Ther. 128, 519–548. 10.1016/j.pharmthera.2010.08.007 20813131

[B23] Clausen B. H. Degn M. Martin N. A. Couch Y. Karimi L. Ormhøj M.. (2014). Systemically administered anti-TNF therapy ameliorates functional outcomes after focal cerebral ischemia. J. Neuroinflammation 11:203. 10.1186/s12974-014-0203-6 25498129 PMC4272527

[B24] Collie A. (2005). Cognition in liver disease. Liver Int. 25, 1–8. 10.1111/j.1478-3231.2005.01012.x 15698392

[B25] Corbalán R. Montoliu C. Miñana M. D. Del Olmo J. A. Serra M. A. Aparisi L.. (2002). Altered modulation of soluble guanylate cyclase by nitric oxide in patients with liver disease. Metab. Brain Dis. 17, 295–301. 10.1023/A:1021953717331 12602506

[B26] de la Torre J. C. Aliev G. (2005). Inhibition of vascular nitric oxide after rat chronic brain hypoperfusion: spatial memory and immunocytochemical changes. J. Cereb. Blood. Flow. Metab. 25, 663–672. 10.1038/sj.jcbfm.9600057 15703700

[B27] Detrait E. R. Danis B. Lamberty Y. Foerch P. (2014). Peripheral administration of an anti-TNF-α receptor fusion protein counteracts the amyloid induced elevation of hippocampal TNF-α levels and memory deficits in mice. Neurochem. Int. 72, 10–13. 10.1016/j.neuint.2014.04.001 24726770

[B28] Dhanda S. Sandhir R. (2015). Role of dopaminergic and serotonergic neurotransmitters in behavioral alterations observed in rodent model of hepatic encephalopathy. Behav. Brain Res. 286, 222–235. 10.1016/j.bbr.2015.01.042 25639545

[B30] D’Mello C. Le T. Swain M. G. (2009). Cerebral microglia recruit monocytes into the brain in response to tumor necrosis factor alpha signaling during peripheral organ inflammation. J. Neurosci. 29, 2089–2102. 10.1523/JNEUROSCI.3567-08.2009 19228962 PMC6666330

[B31] D’Mello C. Riazi K. Le T. Stevens K. M. Wang A. McKay D. M.. (2013). P-selectin-mediated monocyte-cerebral endothelium adhesive interactions link peripheral organ inflammation to sickness behaviors. J. Neurosci. 33, 14878–14888. 10.1523/JNEUROSCI.1329-13.2013 24027287 PMC6705165

[B32] D’Mello C. Ronaghan N. Zaheer R. Dicay M. Le T. MacNaughton W. K.. (2015). Probiotics improve inflammation-associated sickness behavior by altering communication between the peripheral immune system and the brain. J. Neurosci. 35, 10821–10830. 10.1523/JNEUROSCI.0575-15.2015 26224864 PMC6605112

[B29] D’Mello C. Swain M. G. (2014). Liver-brain interactions in inflammatory liver diseases: implications for fatigue and mood disorders. Brain Behav. Immun. 35, 9–20. 10.1016/j.bbi.2013.10.009 24140301

[B33] Edwards T. M. Rickard N. S. (2007). New perspectives on the mechanisms through which nitric oxide may affect learning and memory processes. Neurosci. Biobehav. Rev. 31, 413–425. 10.1016/j.neubiorev.2006.11.001 17188748

[B34] Georgiev P. Jochum W. Heinrich S. Jang J. H. Nocito A. Dahm F.. (2008). Characterization of time-related changes after experimental bile duct ligation. Br. J. Surg. 95, 646–656. 10.1002/bjs.6050 18196571

[B35] Goehler L. E. Gaykema R. P. Hansen M. K. Anderson K. Maier S. F. Watkins L. R. (2000). Vagal immune-to-brain communication: a visceral chemosensory pathway. Auton. Neurosci. 85, 49–59. 10.1016/s1566-0702(00)00219-8 11189026

[B36] Grünhage F. Rezori B. Neef M. Lammert F. Sauerbruch T. Spengler U.. (2008). Elevated soluble tumor necrosis factor receptor 75 concentrations identify patients with liver cirrhosis at risk of death. Clin. Gastroenterol. Hepatol. 6, 1255–1262. 10.1016/j.cgh.2008.06.018 18995216

[B37] Häussinger D. Schliess F. (2008). Pathogenetic mechanisms of hepatic encephalopathy. Gut 57, 1156–1165. 10.1136/gut.2007.122176 18628377

[B38] Häussinger D. Sies H. (2013). Hepatic encephalopathy: clinical aspects and pathogenetic concept. Arch. Biochem. Biophys. 536, 97–100. 10.1016/j.abb.2013.04.013 23643660

[B39] Heldmann U. Thored P. Claasen J. H. Arvidsson A. Kokaia Z. Lindvall O. (2005). TNF-alpha antibody infusion impairs survival of stroke-generated neuroblasts in adult rat brain. Exp. Neurol. 196, 204–208. 10.1016/j.expneurol.2005.07.024 16157335

[B40] Herlenius E. Lagercrantz H. (2001). Neurotransmitters and neuromodulators during early human development. Early Hum. Dev. 65, 21–37. 10.1016/s0378-3782(01)00189-x 11520626

[B41] Huang L. T. Chen C. C. Sheen J. M. Chen Y. J. Hsieh C. S. Tain Y. L. (2010). The interaction between high ammonia diet and bile duct ligation in developing rats: assessment by spatial memory and asymmetric dimethylarginine. Int. J. Dev. Neurosci. 28, 169–174. 10.1016/j.ijdevneu.2009.11.006 19941949

[B42] Huang L. T. Tiao M. M. Tain Y. L. Chen C. C. Hsieh C. S. (2009). Melatonin ameliorates bile duct ligation-induced systemic oxidative stress and spatial memory deficits in developing rats. Pediatr. Res. 65, 176–180. 10.1203/PDR.0b013e31818d5bc7 19047958

[B43] Jain L. Sharma B. C. Srivastava S. Puri S. K. Sharma P. Sarin S. (2013). Serum endotoxin, inflammatory mediators and magnetic resonance spectroscopy before and after treatment in patients with minimal hepatic encephalopathy. J. Gastroenterol. Hepatol. 28, 1187–1193. 10.1111/jgh.12160 23425082

[B44] Javadi-Paydar M. Ghiassy B. Ebadian S. Rahimi N. Norouzi A. Dehpour A. R. (2013). Nitric oxide mediates the beneficial effect of chronic naltrexone on cholestasis-induced memory impairment in male rats. Behav. Pharmacol. 24, 195–206. 10.1097/FBP.0b013e3283618a8c 23591123

[B45] Jiang Y. Deacon R. Anthony D. C. Campbell S. J. (2008). Inhibition of peripheral TNF can block the malaise associated with CNS inflammatory diseases. Neurobiol. Dis. 32, 125–132. 10.1016/j.nbd.2008.06.017 18672064

[B46] Knodell R. G. Ishak K. G. Black W. C. Chen T. S. Craig R. Kaplowitz N.. (1981). Formulation and application of a numerical scoring system for assessing histological activity in asymptomatic chronic active hepatitis. Hepatology 1, 431–435. 10.1002/hep.1840010511 7308988

[B47] Magen I. Avraham Y. Ackerman Z. Vorobiev L. Mechoulam R. Berry E. M. (2010). Cannabidiol ameliorates cognitive and motor impairments in bile-duct ligated mice via 5-HT1A receptor activation. Br. J. Pharmacol. 159, 950–957. 10.1111/j.1476-5381.2009.00589.x 20128798 PMC2829220

[B48] McCoy M. K. Tansey M. G. (2008). TNF signaling inhibition in the CNS: implications for normal brain function and neurodegenerative disease. J. Neuroinflammation 5:45. 10.1186/1742-2094-5-45 18925972 PMC2577641

[B49] Medeiros R. Figueiredo C. P. Pandolfo P. Duarte F. S. Prediger R. D. Passos G. F.. (2010). The role of TNF-alpha signaling pathway on COX-2 upregulation and cognitive decline induced by beta-amyloid peptide. Behav. Brain Res. 209, 165–173. 10.1016/j.bbr.2010.01.040 20122965

[B50] Milewski K. Hilgier W. Albrecht J. Zielińska M. (2015). The dimethylarginine (ADMA)/nitric oxide pathway in the brain and periphery of rats with thioacetamide-induced acute liver failure: modulation by histidine. Neurochem. Int. 88, 26–31. 10.1016/j.neuint.2014.12.004 25523831

[B51] Miller S. L. Wallace E. M. Walker D. W. (2012). Antioxidant therapies: a potential role in perinatal medicine. Neuroendocrinology 96, 13–23. 10.1159/000336378 22377769

[B52] Miralbell J. López-Cancio E. López-Oloriz J. Arenillas J. F. Barrios M. Soriano-Raya J. J.. (2013). Cognitive patterns in relation to biomarkers of cerebrovascular disease and vascular risk factors. Cerebrovasc. Dis. 36, 98–105. 10.1159/000352059 24029412

[B53] Møller S. Bendtsen F. (2015). Complications of cirrhosis. A 50 years flashback. Scand. J. Gastroenterol. 50, 763–780. 10.3109/00365521.2015.1021709 25881709

[B54] Montiel-Castro A. J. González-Cervantes R. M. Bravo-Ruiseco G. Pacheco-López G. (2013). The microbiota-gut-brain axis: neurobehavioral correlates, health and sociality. Front. Integr. Neurosci. 7:70. 10.3389/fnint.2013.00070 24109440 PMC3791857

[B55] Naveau S. Emilie D. Balian A. Grangeot-Keros L. Borotto E. Portier A.. (1998). Plasma levels of soluble tumor necrosis factor receptors p55 and p75 in patients with alcoholic liver disease of increasing severity. J. Hepatol. 28, 778–784. 10.1016/s0168-8278(98)80227-4 9625312

[B56] Odeh M. (2007). Pathogenesis of hepatic encephalopathy: the tumour necrosis factor-alpha theory. Eur. J. Clin. Invest. 37, 291–304. 10.1111/j.1365-2362.2007.01778.x 17373965

[B57] Odeh M. Sabo E. Srugo I. Oliven A. (2004). Serum levels of tumor necrosis factor-alpha correlate with severity of hepatic encephalopathy due to chronic liver failure. Liver Int. 24, 110–116. 10.1111/j.1478-3231.2004.0894.x 15078474

[B58] Riazi K. Galic M. A. Kuzmiski J. B. Ho W. Sharkey K. A. Pittman Q. J. (2008). Microglial activation and TNFalpha production mediate altered CNS excitability following peripheral inflammation. Proc. Natl. Acad. Sci. U S A 105, 17151–17156. 10.1073/pnas.0806682105 18955701 PMC2579393

[B59] Rodrigo R. Cauli O. Gomez-Pinedo U. Agusti A. Hernandez-Rabaza V. Garcia-Verdugo J. M.. (2010). Hyperammonemia induces neuroinflammation that contributes to cognitive impairment in rats with hepatic encephalopathy. Gastroenterology. 139, 675–684. 10.1053/j.gastro.2010.03.040 20303348

[B60] Rosas-Ballina M. Valdés-Ferrer S. I. Dancho M. E. Ochani M. Katz D. Cheng K. F.. (2015). Xanomeline suppresses excessive pro-inflammatory cytokine responses through neural signal-mediated pathways and improves survival in lethal inflammation. Brain Behav. Immun. 44, 19–27. 10.1016/j.bbi.2014.07.010 25063706 PMC4624331

[B61] Schoderboeck L. Adzemovic M. Nicolussi E. M. Crupinschi C. Hochmeister S. Fischer M. T.. (2009). The “window of susceptibility” for inflammation in the immature central nervous system is characterized by a leaky blood-brain barrier and the local expression of inflammatory chemokines. Neurobiol. Dis. 35, 368–375. 10.1016/j.nbd.2009.05.026 19520164 PMC3703512

[B62] Sheen J. M. Chen Y. C. Tain Y. L. Huang L. T. (2014). Increased circulatory asymmetric dimethylarginine and multiple organ failure: bile duct ligation in rat as a model. Int. J. Mol. Sci. 15, 3989–4006. 10.3390/ijms15033989 24603538 PMC3975379

[B63] Sheen J. M. Huang L. T. Hsieh C. S. Chen C. C. Wang J. Y. Tain Y. L. (2010). Bile duct ligation in developing rats: temporal progression of liver, kidney and brain damage. J. Pediatr. Surg. 45, 1650–1658. 10.1016/j.jpedsurg.2009.12.019 20713215

[B64] Shi J. Q. Shen W. Chen J. Wang B. R. Zhong L. L. Zhu Y. W.. (2011). Anti-TNF-α reduces amyloid plaques and tau phosphorylation and induces CD11c-positive dendritic-like cell in the APP/PS1 transgenic mouse brains. Brain Res. 1368, 239–247. 10.1016/j.brainres.2010.10.053 20971085

[B65] Shiraki M. Terakura Y. Iwasa J. Shimizu M. Miwa Y. Murakami N.. (2010). Elevated serum tumor necrosis factor-alpha and soluble tumor necrosis factor receptors correlate with aberrant energy metabolism in liver cirrhosis. Nutrition 26, 269–275. 10.1016/j.nut.2009.04.016 19695831

[B66] Sloan H. L. Good M. Dunnett S. B. (2006). Double dissociation between hippocampal and prefrontal lesions on an operant delayed matching task and a water maze reference memory task. Behav. Brain Res. 171, 116–126. 10.1016/j.bbr.2006.03.030 16677723

[B67] Spengler U. Zachoval R. Gallati H. Jung M. C. Hoffmann R. Riethmüller G.. (1996). Serum levels and in situ expression of TNF-alpha and TNF-alpha binding proteins in inflammatory liver diseases. Cytokine. 8, 864–872. 10.1006/cyto.1996.0115 9047083

[B68] Spiers H. J. Gilbert S. J. (2015). Solving the detour problem in navigation: a model of prefrontal and hippocampal interactions. Front. Hum. Neurosci. 9:125. 10.3389/fnhum.2015.00125 25852515 PMC4366647

[B69] Tain Y. L. Hsieh C. S. Chen C. C. Sheen J. M. Lee C. T. Huang L. T. (2010a). Melatonin prevents increased asymmetric dimethylarginine in young rats with bile duct ligation. J. Pineal Res. 48, 212–221. 10.1111/j.1600-079x.2010.00745.x 20210851

[B70] Tain Y. L. Kao Y. H. Hsieh C. S. Chen C. C. Sheen J. M. Lin I. C.. (2010b). Melatonin blocks oxidative stress-induced increased asymmetric dimethylarginine. Free Radic. Biol. Med. 49, 1088–1098. 10.1016/j.freeradbiomed.2010.06.029 20600827

[B71] Tanida M. Yamamoto N. Morgan D. A. Kurata Y. Shibamoto T. Rahmouni K. (2015). Leptin receptor signaling in the hypothalamus regulates hepatic autonomic nerve activity via phosphatidylinositol 3-kinase and AMP-activated protein kinase. J. Neurosci. 35, 474–484. 10.1523/JNEUROSCI.1828-14.2015 25589743 PMC4293404

[B72] Tau G. Z. Peterson B. S. (2010). Normal development of brain circuits. Neuropsychopharmacology 35, 147–168. 10.1038/npp.2009.115 19794405 PMC3055433

[B73] Teerlink T. Luo Z. Palm F. Wilcox C. S. (2009). Cellular ADMA: regulation and action. Pharmacol. Res. 60, 448–460. 10.1016/j.phrs.2009.08.002 19682580 PMC2767414

[B74] Tobinick E. (2009). Perispinal etanercept for neuroinflammatory disorders. Drug Discov. Today 14, 168–177. 10.1016/j.drudis.2008.10.005 19027875

[B75] Tobinick E. (2010). Perispinal etanercept: a new therapeutic paradigm in neurology. Expert Rev. Neurother. 10, 985–1002. 10.1586/ern.10.52 20518613

[B76] Tobinick E. L. Gross H. (2008). Rapid cognitive improvement in verbal fluency and aphasia following perispinal etanercept in Alzheimer’s disease. BMC Neurol. 8:27. 10.1186/1471-2377-8-27 18644112 PMC2500042

[B77] Tracey K. J. (2002). The inflammatory reflex. Nature 420, 853–859. 10.1038/nature01321 12490958

[B78] Tuttolomondo A. Di Raimondo D. di Sciacca R. Pinto A. Licata G. (2008). Inflammatory cytokines in acute ischemic stroke. Curr. Pharm. Des. 14, 3574–3589. 10.2174/138161208786848739 19075734

[B79] Vallance P. Leone A. Calver A. Collier J. Moncada S. (1992). Endogenous dimethylarginine as an inhibitor of nitric oxide synthesis. J. Cardiovasc. Pharmacol. 20, S60–S62. 10.1097/00005344-199204002-00018 1282988

[B80] Veening J. G. Barendregt H. P. (2010). The regulation of brain states by neuroactive substances distributed via the cerebrospinal fluid; a review. Cerebrospinal Fluid Res. 7:1. 10.1186/1743-8454-7-1 20157443 PMC2821375

[B81] Vélez-Bermúdez I. C. Schmidt W. (2014). The conundrum of discordant protein and mRNA expression. Are plants special? Front. Plant Sci. 5:619. 10.3389/fpls.2014.00619 25426129 PMC4224061

[B82] Wong M. Ziring D. Korin Y. Desai S. Kim S. Lin J.. (2008). TNFalpha blockade in human diseases: mechanisms and future directions. Clin. Immunol. 126, 121–136. 10.1016/j.clim.2007.08.013 17916444 PMC2291518

